# Combining Movement-Related Cortical Potentials and Event-Related Desynchronization to Study Movement Preparation and Execution

**DOI:** 10.3389/fneur.2018.00822

**Published:** 2018-10-05

**Authors:** Hai Li, Gan Huang, Qiang Lin, Jiang-Li Zhao, Wai-Leung Ambrose Lo, Yu-Rong Mao, Ling Chen, Zhi-Guo Zhang, Dong-Feng Huang, Le Li

**Affiliations:** ^1^Department of Rehabilitation Medicine, Guangdong Engineering Technology Research Center for Rehabilitation Medicine and Clinical Translation, The First Affiliated Hospital, Sun Yat-sen University, Guangzhou, China; ^2^Guangdong Provincial Key Laboratory of Biomedical Measurements and Ultrasound Imaging, School of Biomedical Engineering, Health Science Center, Shenzhen University, Shenzhen, China; ^3^Department of Rehabilitation Medicine, The Fifth Affiliated Hospital of Guangzhou Medical University, Guangzhou, China

**Keywords:** electroencephalography analysis, movement-related cortical potential, event-related desynchronization, contingent negative variation, movement preparation, movement execution

## Abstract

This study applied a comprehensive electroencephalography (EEG) analysis for movement-related cortical potentials (MRCPs) and event-related desynchronization (ERD) in order to understand movement-related brain activity changes during movement preparation and execution stage of unilateral wrist extension. Thirty-four healthy subjects completed two event-related potential tests in the same sequence. Unilateral wrist extension was involved in both tests as the movement task. Instruction Response Movement (IRM) was a brisk movement response task with visual “go” signal, while Cued Instruction Response Movement (CIRM) added a visual cue contenting the direction information to create a prolonged motor preparation stage. Recorded EEG data were segmented and averaged to show time domain changes and then transformed into time-frequency mapping to show the time-frequency changes. All components were calculated and compared among C3, Cz, and C4 locations. The motor potential appeared bilaterally in both tests' movement execution stages, and Cz had the largest peak value among the investigated locations (*p* < 0.01). In CIRM, a contingent negative variation (CNV) component presented bilaterally during the movement preparation stage with the largest amplitude at Cz. ERD of the mu rhythm (mu ERD) presented bilateral sensorimotor cortices during movement execution stages in both tests and was the smallest at Cz among the investigated locations. In the movement preparation stage of CIRM, mu ERD presented mainly in the contralateral sensory motor cortex area (C3 and C4 for right and left wrist movements, respectively) and showed significant differences between different locations. EEG changes in the time and time-frequency domains showed different topographical features. Movement execution was controlled bilaterally, while movement preparation was controlled mainly by contralateral sensorimotor cortices. Mu ERD was found to have stronger contra-lateralization features in the movement preparation stage and might be a better indicator for detecting movement intentions. This information could be helpful and might provide comprehensive information for studying movement disorders (such as those in post-stroke hemiplegic patients) or for facilitating the development of neuro-rehabilitation engineering technology such as brain computer interface.

## Introduction

Stroke is one of the most important diseases that threatens human lives and commonly leads to motor impairments in stroke survivors (1). It is essential to understand movement-related brain activity patterns of both healthy people and stroke patients prior to investigating neural recovery mechanisms during stroke rehabilitation processes. Electroencephalograph (EEG)-based electrophysiological technology has been applied in the stroke research area (2–7) in addition to the rehabilitative treatment areas such as motor imagery (MI) and brain computer interface (BCI) (8–13). The aim of this preliminary study was to investigate the different features of movement-related brain activity changes during both motor intentions and motor execution in healthy people, hence facilitating the EEG-based investigational tool in future stroke rehabilitative studies.

Movement-related cortical potentials (MRCPs) and event-related desynchronization/synchronization (ERD/ERS) represent brain activity changes related to movement in the time and time-frequency domains, respectively. Bereitschaftspotential (BP) (14) is the early subcomponent of MRCPs and is believed to be generated by the supplementary motor area (SMA) (15), motor cortex, and cingulate gyrus (14, 16) and represents the motor preparation stage of movement. In addition to BP, a contingent negative variation (CNV) was reported to present in movement preparation stage (17, 18) and reflects motor expectancy and preparation. The late subcomponent, the motor potential (MP), has been proposed to be generated from the underlying motor cortex and partly due to afferents excited by the movement (14). The amplitude of the negativity of MRCPs may relate to the amount of energy required for the movement, while the MRCPs' onset time is interpreted as the length of time taken to plan and prepare the movement (19). Researchers using MRCPs to study motor skill learning found that expert performers showed smaller MRCPs' amplitude and later onset time for the grasped motor skill than beginning learners (19), which indicated that mastery of movement led to reduced energy demand. On the contrary, in cases in which patients with post-stroke hemiparesis performed movements with the paretic limbs, larger MP peak amplitudes were observed, indicating an enhanced energy demand for the lesioned hemisphere (2, 3). ERD refers to the phenomenon of oscillatory components' amplitude decrease resulting from sensory processing or motor behavior (20). Mu ERD is generally regarded as a reliable correlate of increasing cellular excitability in thalamocortical systems during cortical information processing (21) and is observed in motor observation, imagery, preparation, and execution stages. Delayed onset of mu ERD to movement preparation was observed consistently in patients with Parkinson's disease, and patients with somatosensory deficits after stroke showed reduced mu ERD during both movement preparation and actual performance (4, 5). These EEG-based results enhanced the understanding of the mechanisms underlying human movement disorders (14). Since MRCPs and ERD might have different topographical patterns and time course evolution over the movement stages (22), combining these EEG measurements might provide more comprehensive features for understanding movement-related brain functions and detecting movement intentions. Only a few studies have been conducted using the comprehensive EEG analysis method to investigate stroke rehabilitation mechanisms and suggested impairment-specific changes (4). Due to different protocol designs of each study and the small sample size of these studies, more studies combining temporary and oscillatory EEG data to analyze movement-related brain activity changes are necessary for understanding the whole picture of brain motor function.

Motor imagery is a dynamic state of mental rehearsal of movements accompanied by suppression of actual movement (23–25) and might be an alternative treatment for patients with stroke (26). Neuroimaging studies have demonstrated that motor imagery tasks activate brain regions that overlap with brain regions for movement execution (13, 27, 28),and primary motor cortex causes more exchange of causal information among motor areas during a motor execution task than during a motor-imagery task (29). Factors of MI design might affect study outcome, and the first-person perspective kinesthetic-dominant imagery might work better in reorganizing motor-somatosensory networks than other modalities (13). In this study, the ERP protocol created a first-person perspective motor intentions status with inhibition of actual movement, which might help further understanding of MI brain activity changes in electrophysiological aspect.

BCI appears to be a promising technology for helping patients with motor disabilities regain motor control (30, 31). A number of studies have demonstrated the feasibility and possible effects of BCI applied in post-stroke rehabilitation (10, 31–34). However, the technology for detecting motor intention still needs to be refined in order to enhance the accuracy. MRCPs or ERD are indicators commonly chosen to detect the motor intention and results at different accuracy levels (11, 12, 35). Brain activity frequency features seem to yield better accuracy than temporal features in terms of detecting motor intention (35). A study combining MRCPs and ERD/ERS signals for detecting the actual or imagined wrist movement of subjects in different directions reported an average accuracy of 81.5% on the test dataset for two different directions (36). Because of different protocol designs and small samples size of these studies, a better understanding of the movement-related brain activity in time and frequency domains in motor intention stages might add more evidence for optimizing the BCI indicator solution.

In this study, we investigated brain activity changes in both motor intention and motor execution stages in both time and time-frequency domains. Unilateral wrist movements of both arms were involved in this study to investigate the lateralization phenomenon. Several hypotheses were formed: (1) MRCPs and mu ERD would show different stage-specific topographical patterns; (2) brain activities under both movement intention and execution status would show different features in time and time-frequency domains; and (3) MRCPs and mu ERD would distinctly reflect motor intentions and hence, provide precise information for detecting motor intention.

## Methods

### Participants

Thirty-three healthy volunteers (11 males and 23 females) with a mean age of 33.8 (±15.7 SD) years participated in the present study. The subjects were university undergraduate students, postgraduate students, or staff members. The study protocol was approved by the ethics committee of the First Affiliated Hospital of Sun Yat-sen University ([2013]C-068). The inclusion criteria consisted of several parameters: (1) healthy male or female adults; (2) aged 18 years or older; (3) right-handed; (4) interested in this study; and (5) willing to participate in the study as a volunteer, sign the study consent form, and comply with the study protocol. The exclusion criteria consisted of several parameters: (1) individuals with known diseases or conditions that could influence their ability to understand and perform the study tasks. These diseases or conditions included cognitive dysfunction that could affect accurate understanding of study tasks; (2) neural impairments or musculoskeletal disorders affecting upper limb motor function; and (3) other physiological and psychological conditions affecting the ability of accurate understanding and performing the study tasks. All participants were informed of the study protocol, and all questions were explained in detail. Signed informed consent forms were obtained before the study participation. All subjects were self-recognized as right-handed and confirmed by the Edinburgh Handedness Inventory (37) and were ERP study naive.

### Experimental settings and data acquisition

**Figure 1:**
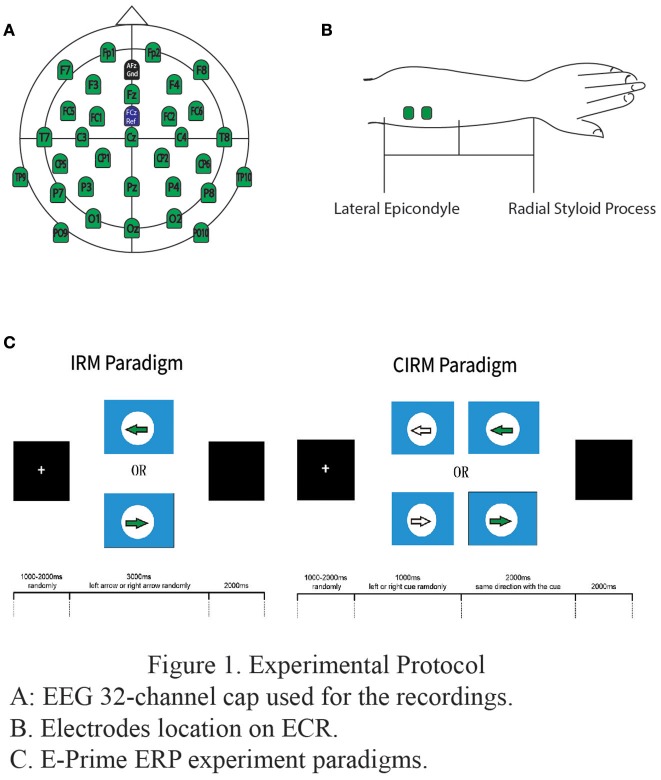
Experimental protocol. **(A)** EEG 32-channel cap used for the recordings. **(B)**. Electrodes location on ECR. **(C)**. E-Prime ERP experiment paradigms.

All experiments in the present study were conducted at the Laboratory of Brain Functional Informatics of Rehabilitation Medicine Department of the First Affiliated Hospital of Sun Yat-sen University in a shielded room, which provided insulation from electromagnetic signals and background noise distractions. Subjects were sitting in front of a table with both forearms resting on the table. A screen was put on the table to present the ERP paradigms at a 75 cm distance from the subjects on the eye level. A BrainAmp 32-channel amplifier from Brain Products (Munich, Germany) was used to record EEG data. Before the experiment, a 32-channel actiCap (Herrsching, Germany) was mounted onto the subject's head, and the Ag/AgCl electrodes were placed according to the extended international 10–20 system, referenced to the FCz and grounded to AFz (Figure 1A). The EEG electrode impedance was kept under 5 kOhm to ensure the quality of EEG recording data, and the sampling rate was 1,000 Hz. Electrooculogram (EOG) was measured by two electrodes, one above the middle point of the right brow to record the EOG vertically and another 2 cm placed aside the outer corner of the right eye in order to record the EOG horizontally.

To measure the movement of wrist extension, EMG electrodes were put on the extensor carpi radialis (ECR) muscles on each side respectively. Before placement of the electrode, the skin was prepared by rubbing slightly with a skin prep gel and cleansing with an alcohol pad in order to reduce the impedance between the electrodes and skin. Bipolar Ag/AgCl round electrodes with a diameter of 2 mm were then put at the middle of proximal half of each forearm, 2 cm apart (Figure 1B). The electrodes were connected to the BrainAmp amplifier in order to synchronize EMG data with EEG data. The EMG electrode impedance was kept under 5 kOhm, and the sampling rate was 1,000 Hz.The single amplifier had an input impedance of 10 MOhm, common mode rejection ratio (CMRR) ≥90 dB, signal-to-noise ratio < 1 μVpp, and actual gain range of ±16.384 mV. The raw EMG was filtered by a Butterworth filter with low and high pass cut-offs of 1,000 and 0.016 Hz, respectively. The raw EMG was stored in the computer for digital processing offline (38). The EMG add-on component of the Brain Vision Analyzer software was used to analyze EMG data offline.

Software E-prime (Psychology Software Tools, Inc, USA) was used to present the visual directions or cues in the study. Subjects were instructed of ERP experiment tasks orally before the tests and in written words on the screen at the beginning of each test. The paradigm of IRM was to present a solid arrow picture pointing either to the left or the right (regarded as “go” signals in this study), and the subjects were requested to perform left or right wrist extension according to the arrow direction. The paradigm started with a white cross in the middle of a black screen as an attention point, which lasted for 800 ms to 1,000 ms randomly. The visual “go” signal pointing to the left or the right was then presented on the screen for a duration of 3,000 ms. The direction was randomly chosen by E-prime, and the arrow picture was followed by a black screen lasting for 2,000 ms. There were 40 trials for each side movement (8). The movement was performed once for each trial, and the subjects rested the arms on the table after the movement until the next required movement in the next trial.

In CIRM, a visual cue (a hollow arrow) containing the forthcoming “go” direction information was added 1 sec before the “go” signal in each trial. The CIRM paradigm began with a white cross in the middle of a black screen and lasted for 800 to 1,000 ms chosen randomly. A visual cue pointing to the forthcoming “go” direction was presented on the screen and lasted for 1,000 ms. After the cue, the visual “go” signal was presented and lasted for 2,000 ms followed by a black screen lasting for 2,000 ms. There were 40 trials for each direction and a total of 80 trials for CIRM. The subject was required to prepare for the movement when seeing the cue and perform the movement upon the presentation of the “go” signal. The movement was performed once for each trial, and the subjects rested their arms on the table after the movement until the next required movement in the next trial (Figure 1C).

Subjects practiced the required movement for 1 to 2 min for each test before the researcher started to record the EEG data. Between the two tests, subjects could take a short break if necessary.

All subjects completed the two tests in the same sequence. EEG, EOG, and EMG data were recorded synchronously by a PC system and analyzed offline later.

### Data processing

All data were analyzed offline using Brain Vision Analyzer 2.1 (Brainproducts, Germany) and Matlab 2014a (The MathWorks Inc., USA). EEG data were re-referenced by the common average of all channels and applied to all channels. Then ICA based Ocular correction was conducted semi-automatically. The Ocular components were classified by the Analyzer and confirmed by the researchers, then removed from the EEG data. A band-filter was applied with a low cutoff at 0.01 Hz (12 decimal/octave), and high cutoff at 50 Hz (12 decimal/octave). Laplacian method was used to remove volume conduction effects which lead to low spatial resolution (39). It has been proved that the Laplacian method, compared with other fixed spatial filter, such as bipolar and common average reference, can effectively improve the Signal-Noise-Ratio of the EEG signal for the ERD/ERS analysis (40). By subtracting the mean activity at surrounding electrodes from the channel of interest, a finite difference method is used to calculate the Laplacian derivations. EEG data were segmented from −1.2 to 3.0 s relative to the GO signal for both test. IRM EEG data were baseline corrected using −1.2 to 0 s as the baseline. While CIRM EEG data were using −1.2 to −1.0 s as the baseline since the visual cue was presented at the time of −1.0 s. Trials of movement sessions of each side were averaged within-subjects, and grand averaged among all subjects later. The peak information of MP and CNV was detected by BrainVision Analyzer and exported for statistics analysis. Matlab 2014a was used to calculate the time-frequency mapping and mu ERD. The segmented EEG data were transformed with an averaged Continuous Wavelet Transformation (CWT). The 8–12 Hz frequency band data were abstracted from the time-frequency mapping and transformed into mu ERD graph. The EMG activity onset for each trial was determined by crossing the threshold at 4 SD from baseline mean (3) in either direction. EMG peaks were detected by the Brain Vision Analyzer and exported with time and amplitude information.

### Statistical analysis

EMG onset and peak times and EMG peak value were compared between sides by *t*-test and compared between IRM and CIRM by paired sample *t*-test. The MP component in both tests and the CNV component in CIRM were calculated at C3, Cz, and C4 locations. All component information, including latency and each wave's amplitude, were compared among locations and sides by the one-way multivariate analysis of variance (one-way MANOVA). The MP latencies of IRM and CIRM were compared by paired sample *t*-test. The mean values of the time-frequency response were compared among C3, Cz, and C4 locations and among sides and movement stages by one-way MANOVA. To test the contra-lateralization feature of mu ERD and MRCP in motor intention/preparation stage, paired-sample *t*-tests of mu ERD mean values and CNV amplitudes among C3, Cz, and C4 location during unilateral movement preparation stage were performed. All statistical analyses was conducted with software IBM SPSS statistics 21. Statistical significance was set at 0.05 for all tests.

## Results

### EMG

**Figure 2:**
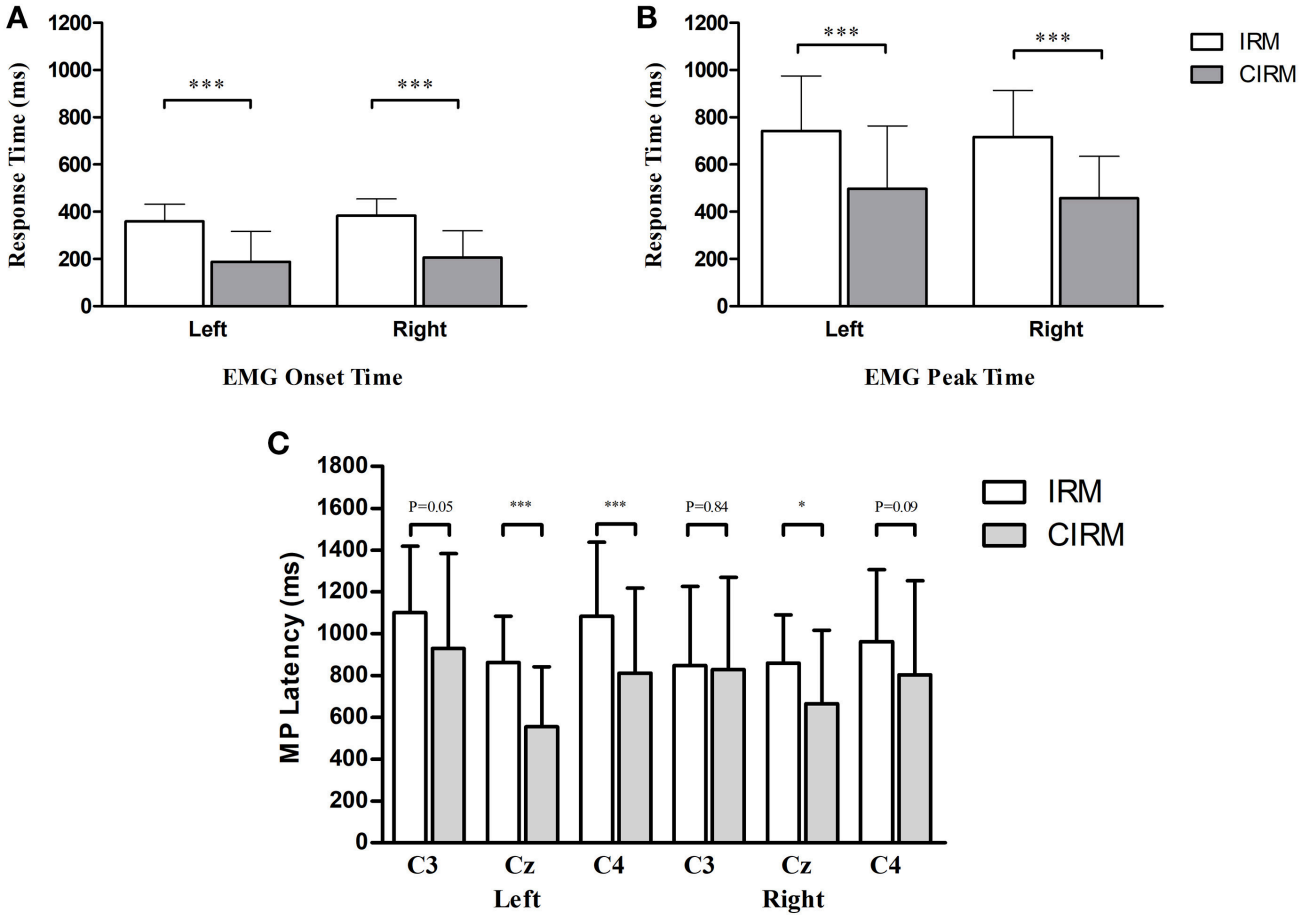
Bar graphs showing averaged EMG onset time **(A)**, EMG peak time **(B)**, and MP latency **(C)** in IRM and CIRM. Error bars represent the SD. Significant differences are indicated by asterisks. **(A)** The average EMG onset time is shorter in CIRM than in IRM. **(B)** The average EMG peak time is shorter in CIRM than in IRM. **(C)** The MP latency was shorter in CIRM than in IRM at Cz during both side movements and at C4 during left wrist movement. *p < 0.05, ***p < 0.001.

There were no significant differences found between right and left wrist movements with respect to average EMG onset and peak times and EMG peak value in both tests. There were significant differences of the EMG onset and peak times between IRM and CIRM with both left and right wrist movements (*P* < 0.01). The EMG onset and peak times were shorter in CIRM than in IRM for both right and left wrist movements (*P* < 0.01) (Figure 2). There was no significant difference in the EMG peak value between IRM and CIRM.

### MRCP and CNV

**Figure 3:**
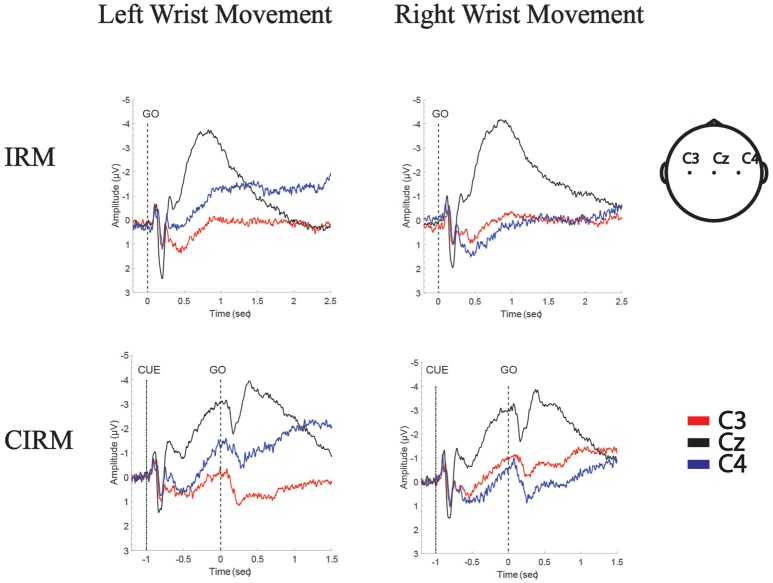
Grand Average of MRCPs of all subjects in IRM and CIRM. MP is presented in movement execution stage of both tests, and CNV is presented in movement preparation stage of CIRM. Negative Upwards.

**Figure 4:**
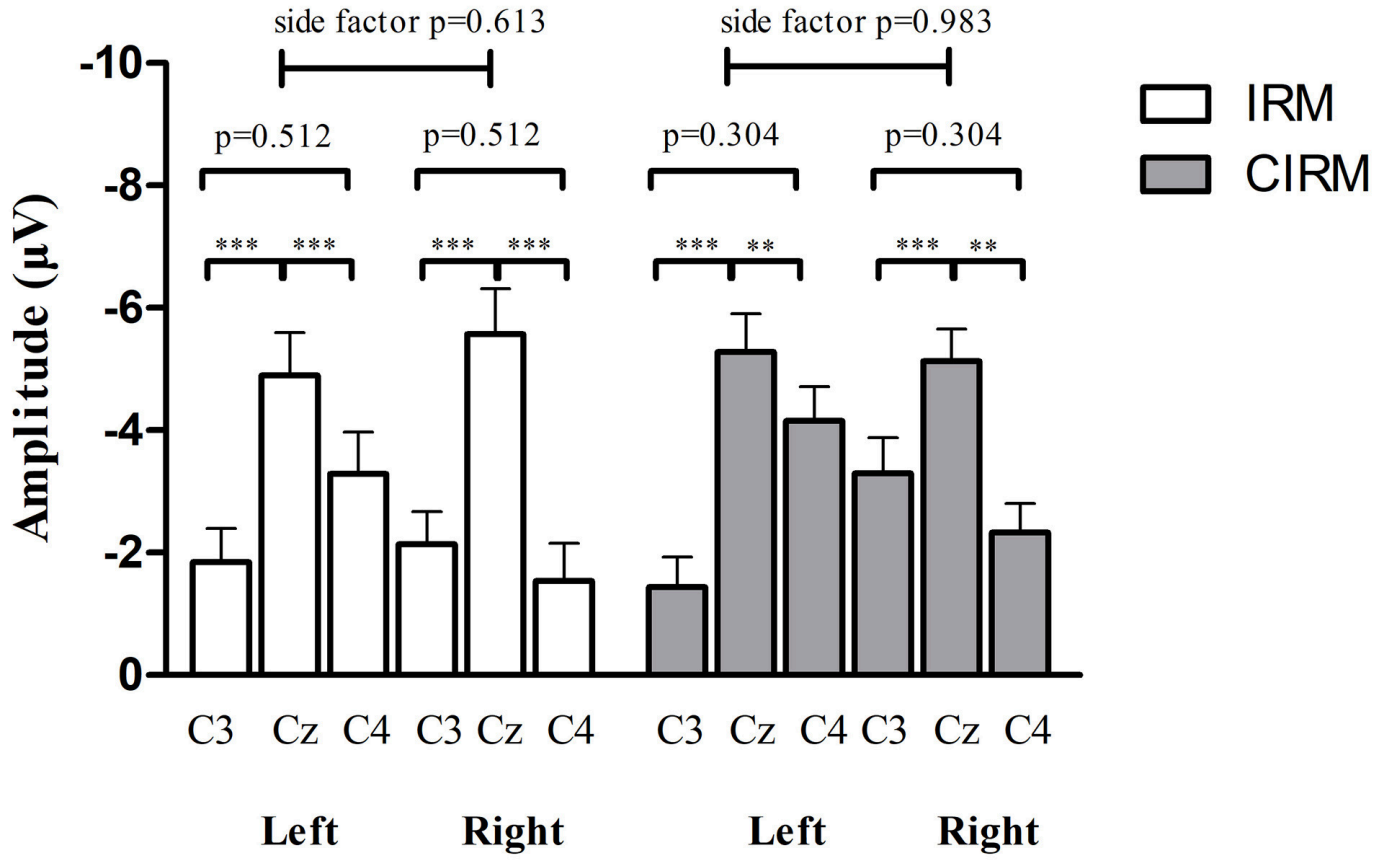
MP amplitude at C3, Cz, and C4 location in IRM and CIRM. There were significant differences between Cz and C3 and between Cz and C4 (*p* < 0.01), but not between C3 and C4 in both tests. Negative upwards. **p < 0.01, ***p < 0.001.

In the movement execution stage of both tests, the MP appeared bilaterally, with the largest peak at Cz (Figure 3). A One-way MANOVA was conducted to assess the differences in MP amplitude among different locations and on arm side movements. The multivariate effect was significant by locations in IRM and CIRM (*F* = 15.069, *p* < 0.01 and *F* = 14.248, *p* < 0.01, respectively). The *post-hoc* tests showed significant differences between Cz and C3 or Cz and C4 (*p* < 0.01) but not between C3 and C4 in both tests (Figure 4). The paired-sample *t*-test showed that there were significant differences in MP latencies between IRM and CIRM at Cz during both side movements and at C4 during left wrist movement (*p* < 0.01) (Figure 2). However, no significant differences were found in MP amplitude between IRM and CIRM tasks in all three locations.

In CIRM, the visual cue divided the task into prolonged movement preparation and movement execution stages. A CNV wave presented during the movement preparation stage, peaking shortly after the “go” signal followed by the MP (Figure 3). One-way MANOVA analysis showed no significant multivariate effects on locations or sides. To test the contra-lateralization feature of CNV, a paired-sample *t*-test of CNV amplitudes between C3 and C4 locations was performed and showed no significant differences.

### Time-frequency mapping and mu ERD

**Figure 5:**
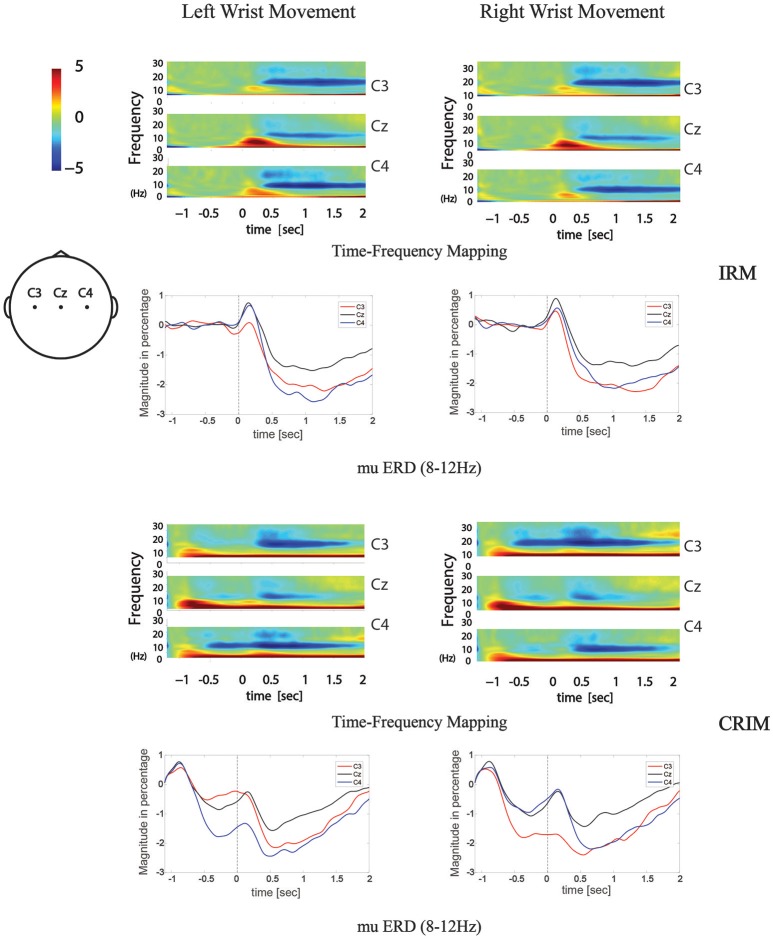
Time-frequency mapping and mu ERD during movement tasks in both tests. Frequency data was transformed from grand average EEG data of all subjects. 8–12 Hz frequency band data was abstracted from the time-frequency mapping and transformed into the mu ERD line graphs.

The time-frequency mapping and mu ERD calculated from the total average EEG data from all subjects are illustrated in Figure 5. The mean values of the time-frequency response in the 8–12 Hz mu rhythm band were compared among by one-way MANOVA. In IRM, there were two factors: (1) locations (C3, Cz, and C4) and (2) movement sides. No significant multivariate effects were found on locations or sides (*F* = 1.541, *p* = 0.217 and *F* = 0.116, *p* = 0.864, respectively). In CIRM, there were three factors: (1) locations; (2) sides; and (3) movement stages (movement preparation and execution). Significant multivariate effects were found with respect to stages (*F* = 5.920, *p* = 0.015) but not for locations (*F* = 2.642, *p* = 0.072) or sides (*F* = 0.226, *p* = 0.635).

To test the contra-lateralization feature of mu ERD, the mean values of the time-frequency response in the 8–12 Hz mu rhythm band were compared among different locations by paired-sample *t*-test. During movement execution stage of both tests, there were no significant differences found between C3 and C4. During the movement execution stage of right wrist extension, there were significant differences found between C3 and Cz or C4 and Cz (*P* < 0.05). During the movement execution stage of left wrist extension, there were significant differences found between C4 and Cz (*P* < 0.05).

**Figure 6:**
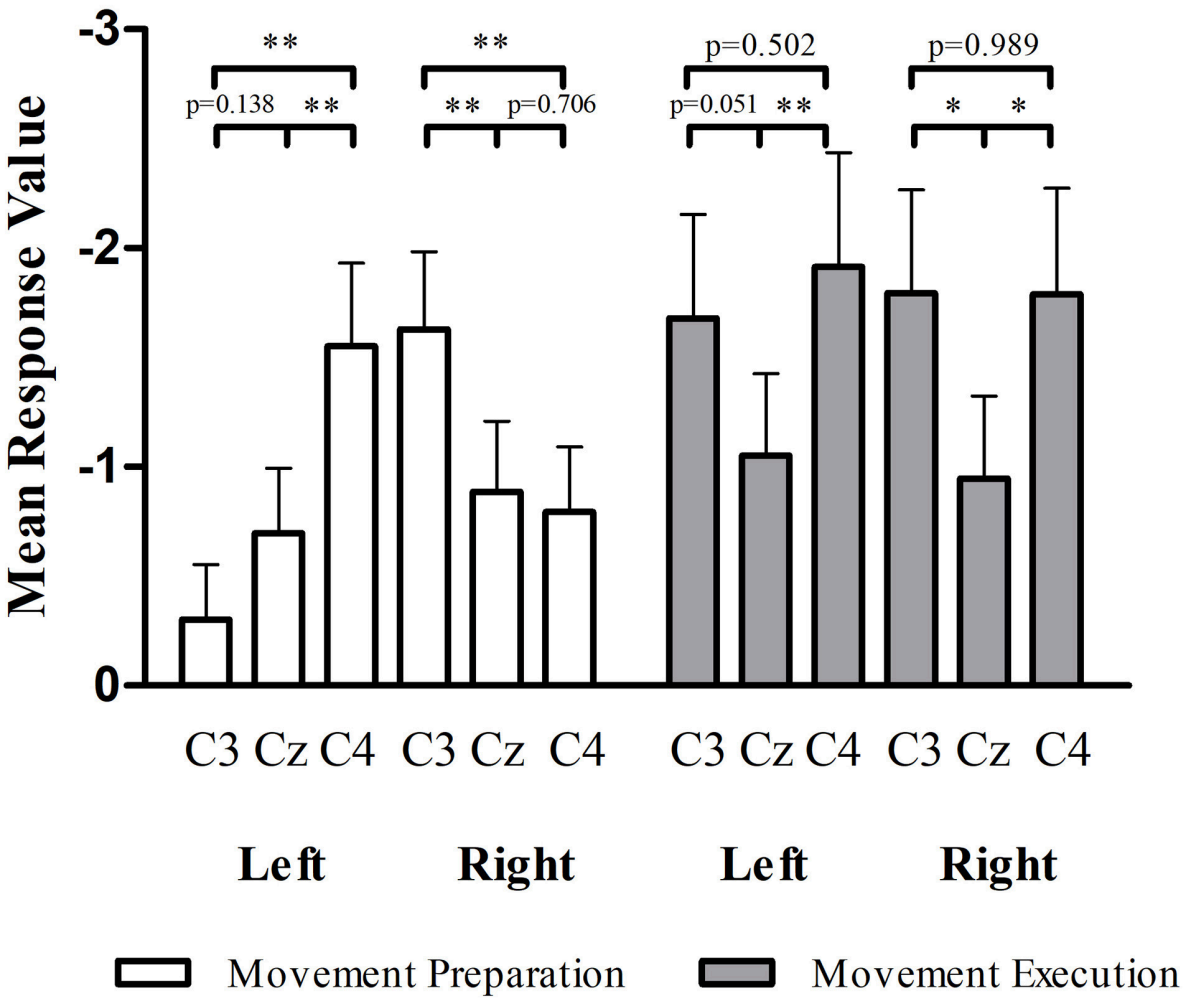
mu ERD at C3, Cz, and C4 in movement preparation and movement execution stage of CIRM. In the movement preparation stage, the contralateral sensory motor cortex (C4 for left wrist movement, C3 for right wrist movement) showed larger mu ERD than other locations (*p* < 0.05). In the movement execution stage, Cz showed the lowest amplitude of mu ERD and no significant differences found between C3 and C4. *p < 0.05, **p < 0.01

In the preparation stage of CIRM, mu ERD showed significant differences between C3 and C4 during both left and right wrist movements (*p* = 0.001 and *p* = 0.005, respectively). There were significant differences found between Cz and the contralateral sensory motor cortex area (C3 for right wrist movement, *p* = 0.006, C4 for left wrist movement, *p* = 0.001). Different from the phenomenon of Cz that showed the largest amplitude of MP and CNV, the mu ERD showed the least amplitude in Cz among the three locations (Figure 6).

## Discussion

In the present study, we recorded and analyzed brain activity changes in the time and frequency domains during both motor preparation and execution stages. IRM used a motor response paradigm with visual picture instructions, which made a subject perform the required movements in a more impulsive manner compared to self-initiated movements (41). CIRM used a visual cue contenting the forthcoming movement information 1 sec before the “go” signals, which divided the motor task into two clearly separated periods (42). During the 1 sec from the cue to the “go” signal, the brain activity was under a state of motor intention, expectation, and preparation condition.

The EMG onset and peak times were shorter in CIRM rather than in IRM for both side movements, and the MP latency in CIRM was shorter than in IRM. This result confirmed the commonly observed phenomenon that given a cue and extra preparation stage, people respond to the “go” signal faster (Figure 2). The visual cue containing information relevant to the movement might combine the sensory stimuli responses with sensorimotor area and facilitate movement initiation. The effects of external cues were observed in previous studies and proved to be beneficial for speeding movement of patient with motor deficits (43, 44).

Generally speaking, in the time-voltage change domain, MP reflected the brain activity changes during the movement execution stage (45) while the CNV represented movement intention, expectancy, and preparation in the pre-movement stage (42, 46). In the movement execution stage of both tests in current study, MP amplitude showed no significant differences between C3 and C4, indicating that the movement execution involved the sensorimotor cortex bilaterally. Components of MRCP were proposed to be generated by summed excitatory postsynaptic potentials in apical cortical dendrites (47), which might be an explanation for the largest MP amplitude recorded at Cz. Bilateral excitatory postsynaptic potentials were transferable, and the central area (Cz) gathered the highest sum of potentials. CNV is a complex ERP component related to the cognitive process of stimulus anticipation. Although it was usually investigated with paradigms with cues contenting information differentiate from the “go” signal in previous studies, CNV was observed to be evoked with cues containing similar information to the “go” signal in this study and may mainly contribute to the movement expectancy and motor preparation to the forthcoming movement (42). In the movement preparation stage, CNV also showed a bilateral control phenomenon and the highest peak at Cz.

From the time-frequency mapping and mu ERD data results, we could observe bilateral control phenomenon in the movement execution stage of both tests. However, different from the MRCP findings, the amplitude of frequency changes was lowest at Cz (Figure 5). Since mu ERD was regarded as a reflection of brain activation (48) and has a maximum correlation with sensorimotor cortex area (49), we could assume that it has less transferable features than the cortex potentials, hence, it represents brain activity changes more precisely in the topographical view. In the movement intention/preparation stage, the mu ERD appeared mainly contralaterally, indicating that the brain excitability has a contralateral feature in the pre-movement period. Starting from the contralateral Rolandic region and becoming bilaterally symmetrical with the execution of movement, the mu ERD feature presented in this study was consistent with those from previous studies (50).

**Figure 7:**
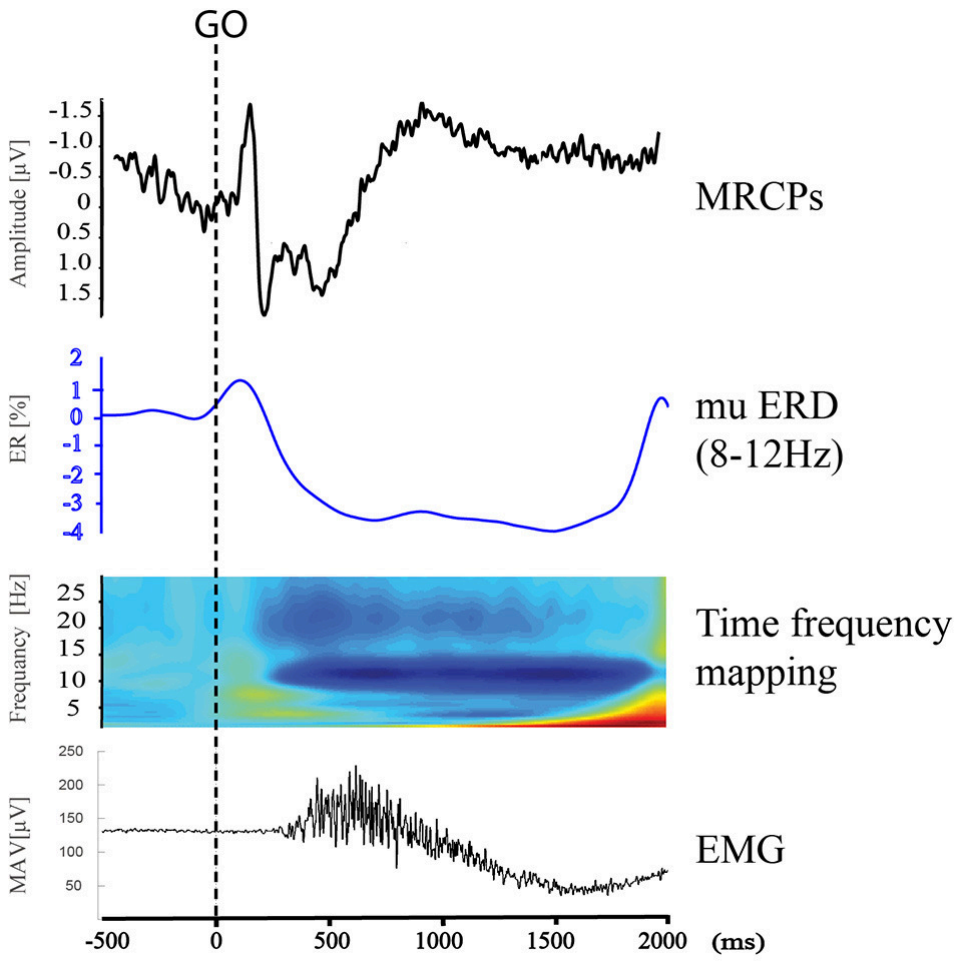
MRCPs, mu ERD, time frequency mapping at C3, and right upper limb EMG during Right Wrist Extension in IRM.

Combining EEG changes in the time and time-frequency domains during pre- and movement execution stages, we could see that the time courses of MRCP and mu ERD were aligned (Figure 7) and consistent with previous studies regarding movement-related brain activity changes (51), while each indicator showed different topographical features. This might suggest different neuronal mechanisms between the generation of the two phenomena (22). MRCPs mainly represent an increase in task-specific responses of the SMA and contralateral M1-S1 and partly reflect the afferents' excitation caused by the movement, while mu ERD reflects changes in the brain activation level such as cortical sensorimotor area involved in movement intention, expectancy, preparation, and execution periods. Taken together, the MRCPs and mu ERD structured a dynamic involvement of human primary, supplementary motor, and sensorimotor cortices for movement planning and execution (51). The combined EEG analysis method could provide more comprehensive information for the investigation of movement disorders such as the energy demanding and consuming information from MRCPs (52) and the brain activation information from mu ERD (48, 53, 54).

Although the lateralization phenomena differed in MRCP and mu ERD features, it was clear that both contralateral and ipsilateral motor cortices were involved in motor tasks. Neural networks within and between hemispheres are necessary to coordinate motor functions not only for bilateral but also for unilateral movements. The neural network generating the coordination function between hemispheres was proposed to be the cingulate motor area and the cerebellum (55). The ipsilateral hemisphere involved in a motor task might contribute to the modulation, balance, or prohibition of the motor tasks. The present ERD results suggested that this bilateral movement coordination was mostly involved in the motor execution stage (Figure 5). The ipsilateral sensorimotor cortex might be an integral part of motor regulation (56). In the movement intention and preparation conditions, the oscillatory brain components showed stronger contralateral dominancy. This feature might have special value in investigational and clinical applications. Specifically, mu ERD could be a more sensitive indicator than MRCPs for BCI technology in order to detect movement intentions (8, 10, 49), especially if the direction information of movements are among study interests (36).

There were some limitations to the present study. The first one was that the initial part of MRCPs was mixed with a Visual Evoked Potential after visual stimuli in both paradigms, and it could not be precisely used to identify the onset of the BP component of the MRCPs. Hence only MP peak information was calculated and analyzed in this study. Another limitation of the study was that only right-handed subjects were recruited in this study. In the previous study, the contralateral preponderance of mu ERD was studied and showed a difference between right-handed and left-handed subjects (50). However, this phenomenon was not studied in this program with only right-handed subjects.

## Conclusion

This study investigated brain activity changes during movement intention, preparation, and execution stages of wrist extension using the comprehensive EEG analysis method, which combined indicators such as MRCPs, CNV, time-frequency mapping, and mu ERD. EEG changes in the time and time-frequency domains showed different topographical features and might provide comprehensive information for studying movement disorders such as those in post-stroke hemiplegic patients. Additionally, movement execution was controlled bilaterally, while the movement intention was controlled mainly contralaterally by sensorimotor cortices. Mu ERD was found to have stronger contra-lateralization features in the movement intention stage and might be a better indicator for detecting movement intentions in neuro-rehabilitation engineering technology such as BCI.

## Ethics statement

The study was approved by the Human Subjects Ethics Sub-committee of the First Affiliated Hospital, Sun Yat-sen University, China (ethic number [2013]C-068).

## Author contributions

HL and GH analyzed the data and interpreted the results. HL drafted the manuscript. HL, QL, J-LZ, W-LL, Y-RM, and LC conducted the experiment and collected the data, Z-GZ conducted part of the data analysis and interpreted the results. HL and GH contributed to the revision of the Manuscript and the answers to the comments from the reviewers and editor. D-FH and LL designed the study and supervised all stages of the study including data collection, analysis, interpretation, and substantial revision of the manuscript. All the authors approved the final version of the manuscript.

### Conflict of interest statement

The authors declare that the research was conducted in the absence of any commercial or financial relationships that could be construed as a potential conflict of interest.
